# Elastic Intramedullary Nails in the Treatment of Multi-Segmental Humeral Fracture in a Polytrauma Patient

**DOI:** 10.7759/cureus.16161

**Published:** 2021-07-04

**Authors:** Konstantinos Sidiropoulos, Efstratios D Athanaselis, Alexandros Saridis, Alexios Agapidis, Alkis Saridis

**Affiliations:** 1 Orthopaedics, General Hospital of Serres, Serres, GRC; 2 Orthopaedics, University of Patras, Patras, GRC; 3 Orthopaedic Surgery and Musculoskeletal Trauma, University Hospital of Larissa, Larissa, GRC; 4 Orthopaedics, General Hospital of Drama, Drama, GRC; 5 Orthopaedic Surgery, General Hospital of Serres, Serres, GRC

**Keywords:** polytrauma patient, multi-segmental humeral fracture, elastic intramedullary nails, upper extremity fracture, treatment option

## Abstract

A polytrauma patient with a life-threatening condition is a quite demanding situation due to special considerations regarding the time, the way, and the sequence of the necessary procedures. Elastic intramedullary nails (EIN) could be used under these conditions for adult patients with humeral fractures requiring fixation. Here we present a case of a multi-segmental closed humerus fracture in a polytrauma patient treated by EIN. The general condition of the patient and the need for multiple surgical procedures required the selection of a minimally invasive and time-saving fixation technique. The patient’s follow-up was uneventful with complete healing of the humeral fracture; the functional scores results are excellent at five years post operatively. A review of the literature revealed limited published cases of humeral fractures (128 patients) treated by EIN, despite the fact that the results are quite encouraging. Based on our results and the current literature, we believe that EIN could become an effective treatment choice for multi-level humerus fractures, especially in damage control surgery.

## Introduction

The fixation method of fractures and the duration of surgical procedures are crucial parameters for the survival of polytrauma patients [1]. Elastic intramedullary nails (EIN) [2], which are commonly used in the treatment of fractures in children and adolescents, could be the ideal choice of treatment for long bones fractures in damage-control surgery. Elastic nails are applied in a minimally invasive way, quickly and are suitable for final treatment. EINs are made of titanium with one pre-bent edge and resistant to plastic deformation. The more proximal end to the surgeon is smooth and cylindrical allowing hammering of the nail while the distal end is sharp and sphenoid in order to make intramedullary advancement of the nail, easy. Fixation of the nail is succeeded by its elastic deformation as long as its initial curvature is reduced within the medullary canal of the long bone achieving what is called a 3-point stabilization. However, axial and rotational stability of the fracture is obtained by the application of at least two EINs. Nails must fit in most of the medullary canal in order to maintain the rotational and tensile stability [1] and their appropriate width must be estimated preoperatively [3].

Retrograde or antegrade insertion of the nail can be performed. Proximity of the fracture should be taken into account by selecting the more proximal end of the bone for insertion. To rephrase, the smaller the distance between the entry point and fracture line, the easier the reduction. Image intensifier and radiolucent table are essentials for this procedure. According to the type of fracture and the stability achieved, a splint can be used. EINs applied by proper technique under strict indications (proper nail length and width, proper entry point, and use of at least two nails) could be added into the arsenal of orthopedic surgeons for the challenging treatment of trauma patients. This is the aim of the present study, along with a literature review that justifies our point of action.

## Case presentation

**Figure 1:**
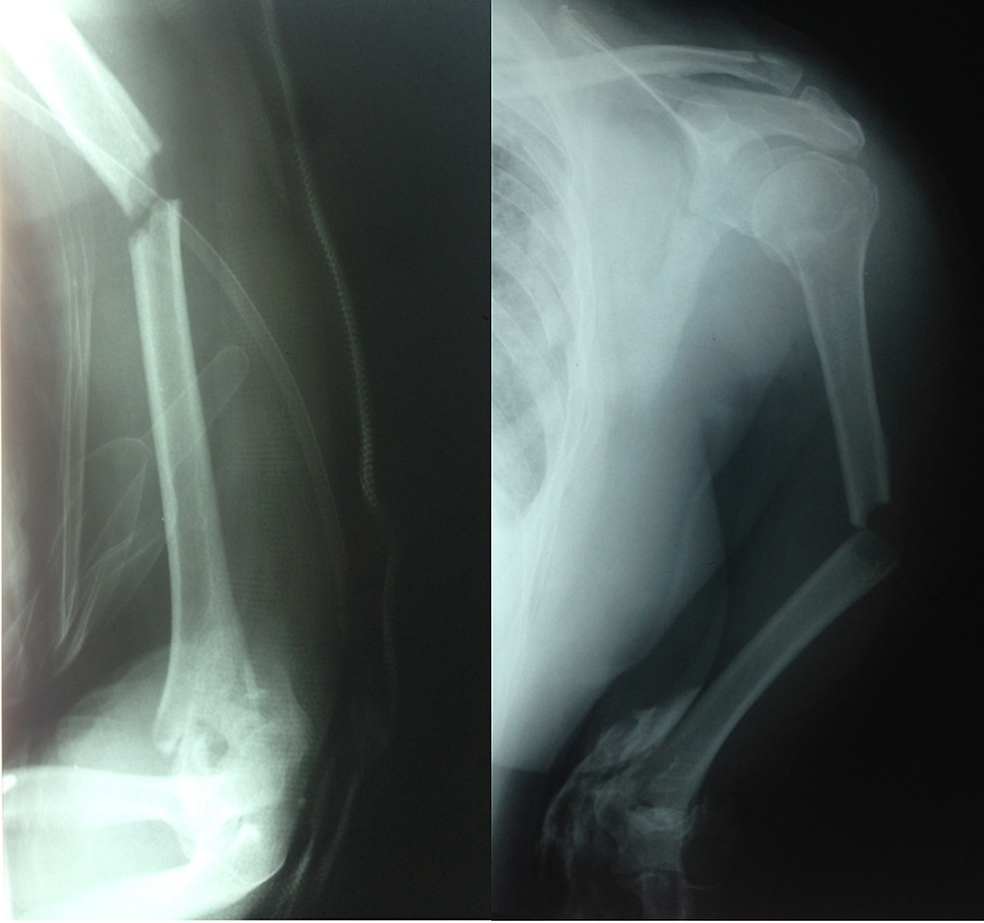
Preoperative X-rays where the proximal humerus, the middle shaft, and the supra condylar fracture are seen

A 57-years-old female was admitted to the emergency department due to a motor vehicle accident. The patient suffered from multiple severe injuries. After clinical and radiological evaluation, a moderate cerebral contusion (GCS 11), multiple rib fractures with pneumothorax, right supracondylar femoral fracture (AO classification type C3), left ankle fracture (AO classification type C) and a segmental left humerus fracture were diagnosed. Humeral injury included a sub-capital fracture (type 11B1), a shaft fracture (type 12A2b) and a supracondylar fracture (type 13A3.3) (Figure 1). A distal undisplayed clavicle fracture (type 151A) and a fracture of the body of scapula were treated conservatively.

**Figure 2:**
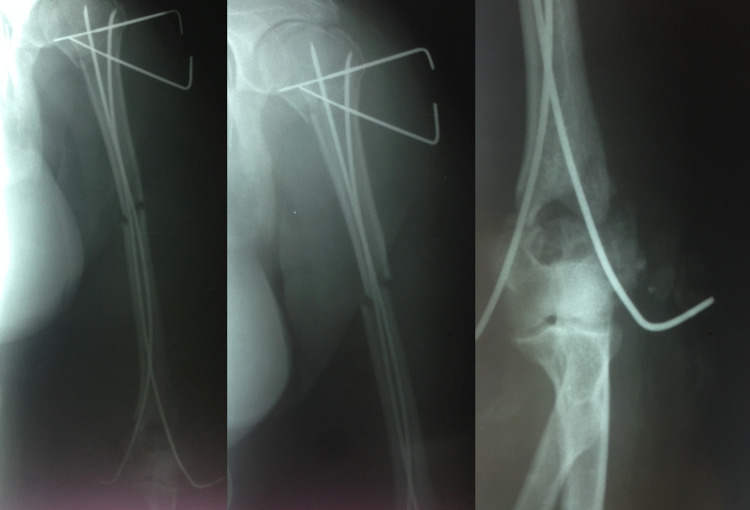
Immediately postoperative X-rays of the humerus - Kirschner wires were induced percutaneously with the outer edge outside of the body and it remained there for four weeks

Neurovascular status of the injured limbs was normal [4]. After the initial evaluation, and expertise trauma unit coped with the pneumothorax using Büllau thoracic pipe and immobilized fractured limbs with splints according to the advanced trauma life support (ATLS) protocol. The patient was hospitalized in the ICU and six days later, definite surgical treatment of fractures was performed. Intramedullary nailing of the femur and open reduction with internal fixation of the ankle fracture took place initially. In order to avoid the risks of a long-lasting operation, EIN was selected for the humeral shaft and supracondylar fractures fixation, together with two Kirschner wires (K-wire) 2.0 for the sub-capital fracture which was induced percutaneously after closed reduction (abduction and internal rotation) (Figure 2). Image intensifier and radiolucent table were used for this procedure.

**Figure 3:**
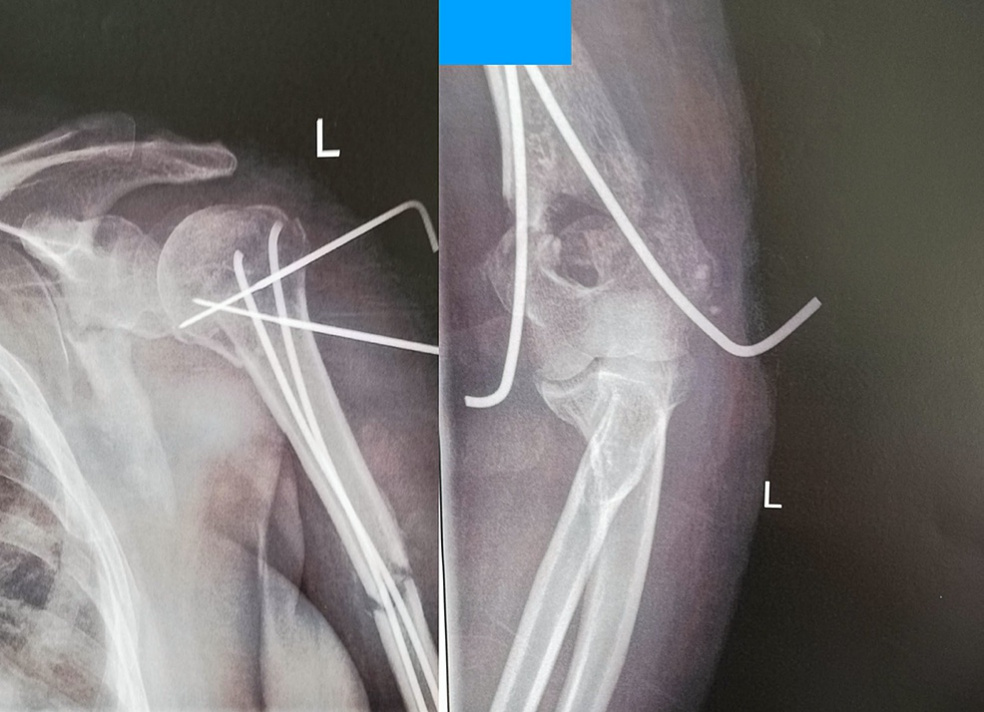
Four weeks postoperatively - the two Kirschner wires were removed the following day

**Figure 4:**
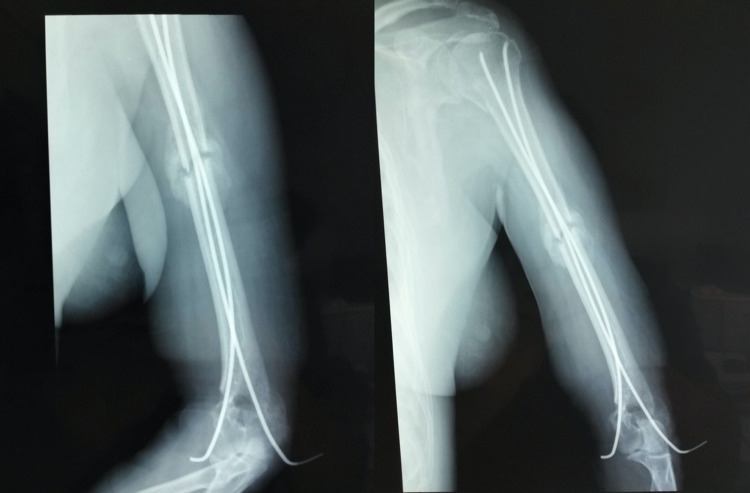
Three months postoperatively - the patient was able to passively move the upper limb without pain

**Figure 5:**
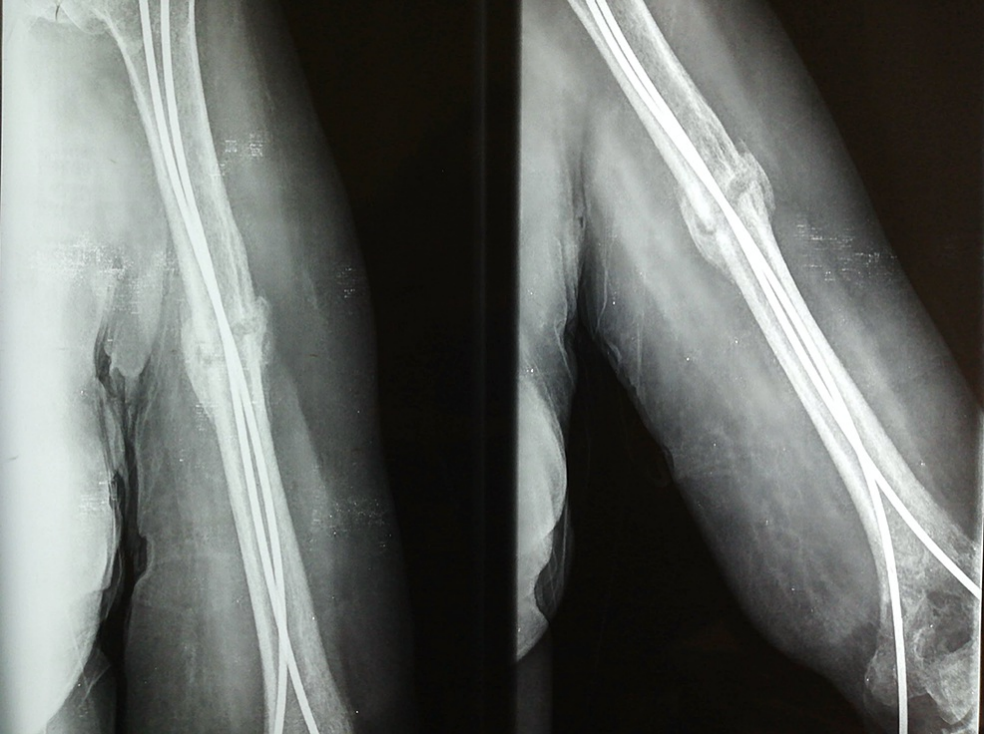
Fourteen weeks postoperatively - the patient was encouraged to perform active abduction and freely move her elbow

**Figure 6:**
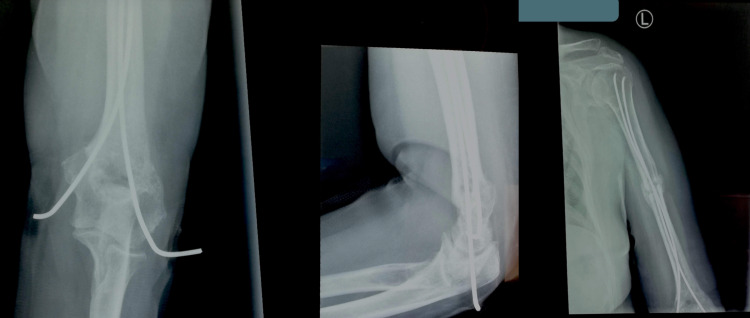
Four months postoperatively - the fractures healed and the elastic stable intramedullary nailings were removed

Two 2-cm long skin incisions and a hole with a 2.5 mm drill on bone cortex were made over medial and lateral humeral epicondyle and a nail was introduced from each insertion point into the medullary canal. The two elastic nails were then, advanced by rotational movements, taking care to pass the fracture site by keeping the fracture reduced and the bone aligned under fluoroscopy. The tip of each nail was anchored to the cancellous bone of proximal humeral metaphysis. A long arm splint was applied for four weeks. The patient was discharged from the ICU after 20 days and afterwards, treatment was continued at a rehabilitation centre for two months. During the postoperative follow-up period, regular radiological evaluation took place (Figures 3-6).

**Figure 7:**
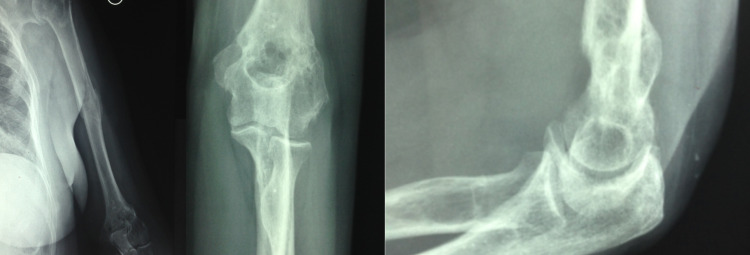
Six months postoperatively - the patient was able to perform nearly every daily activity without pain; rarely used NSAIDs for night pain NSAIDs: nonsteroidal anti-inflammatory drugs

**Figure 8:**
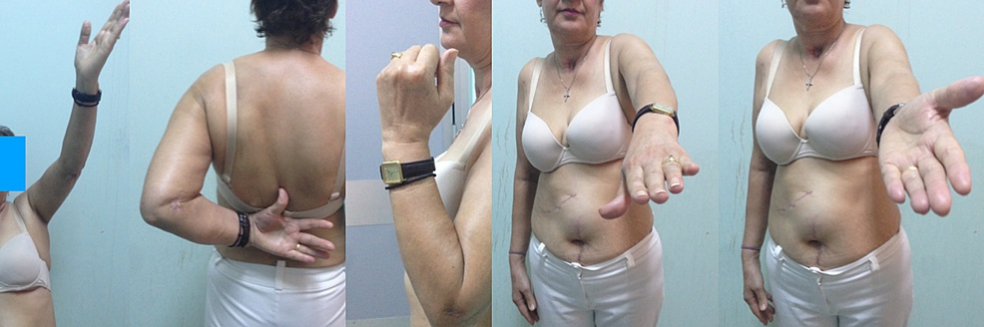
The same day as above, during the clinical examination; the patient was in no pain at all

There was no neurovascular deficiency. Elastic nails were removed from the humerus four months postoperatively after clinical and radiological evidence of the fractures’ consolidation, and a month later, the patient was able to have almost a full range of motion of her shoulder and elbow (Figures 7-8). Pendulum exercises of the shoulder had begun after the removal of the K-wires. Simultaneously, the elbow’s passive flexion and extension up to the pain limit was encouraged. Active exercises and muscle strengthening were commenced after the third post-operative month. 

**Figure 9:**
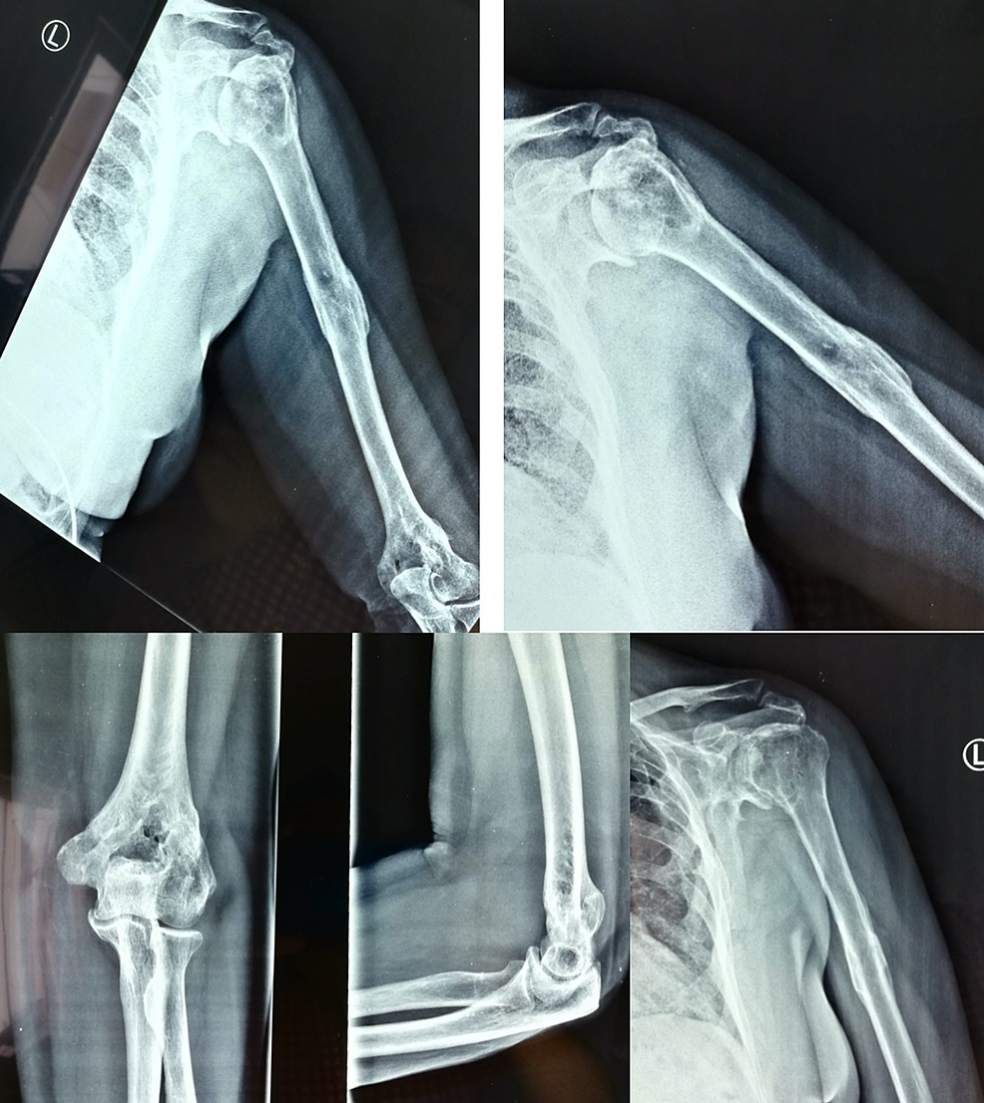
Fifty-five months postoperatively - painless and full range of motion with excellent functional shoulder and elbow scores; no reduction to grip strength

The patient has a full range of movement in her left upper limb joints and is absolutely pain-free 55 months after the accident (Figure 9). She copes with daily-living activities except for over-head lifting of heavy objects (above 10 kg), and pushing a heavy object (more than 40 kg) with one hand. The patient's disabilities of the arm, shoulder, and hand (DASH) score was 2.5/100 and the American Shoulder and Elbow Surgeons Standardized Shoulder Assessment Form (ASES) score was 97/100 [5-7].

## Discussion

Segmental humeral fractures are rare and limited literature is available concerning their classification and treatment. The co-existence of these three different types of fractures extends surgical approaches, increases tissue damage and surgical time, thereby increasing non-union and infection rates [8-12].

In the case of polytrauma patients, damage-control surgery precludes long-lasting surgical procedures. Moreover, definite fracture treatment after initial stabilization (as soon as the general condition of the patient allows for it) may include multiple surgical interventions for different anatomic regions, increasing the duration of the operation and the patient’s peri-operative stress. Therefore, the orthopedic surgeon often has to select minimally invasive methods for definite treatment.

Our polytrauma patient had a multi-segmental humeral fracture, combining the aforementioned fracture locations. Non-operative treatment was not a choice because of significant axial and rotational instability. Any device of external fixation would fail as a possible definite treatment. A combination of plates would need extended approaches, increasing periosteal stripping and elongating the duration of surgery. These parameters are well-known causes of increased non-union and infection rates. Additionally, multiple plates applied on the long bone are a risk factor for post-operative peri-implant fractures due to the effect of increased stress.

Thus, a minimally invasive fixation technique by EIN and additional K-wires was selected. Two elastic nails were inserted from medial and lateral epicondyle in a retrograde manner, cross-passing supracondylar and diaphyseal fracture, and two K-wires stabilized sub-capital fracture percutaneously. However, the effectiveness of elastic nails in preventing rotational instability is limited. The elasticity of the nail curve, anchoring in proximal and distal metaphyseal bone and cortex, and the use of more than one nail, provide adequate axial and angular stability but rotational forces, applied by forearm movements cannot be successfully neutralized. Therefore, a long arm splint was used for 3-4 weeks post-operatively and care was taken to avoid rotational forces on the arm during physiotherapy exercises of shoulder and elbow.

Zatti et al. (1998) published a retrospective study comparing intramedullary nailing in 14 cases using the fixed type of EIN (Marchetti-Vicenzi) with plate osteosynthesis (30 cases). Results of elastic nailing concerning fracture healing and functional rehabilitation were equal to plating with fewer and milder complications [2]. Analogous results have been presented by Williams and Shewring [13] using the Marchetti-Vicenzi type of EIN nail in seven patients (male and female) between 15 to 91 years of age, achieving union in six weeks with the exception of one case in which the healing process lasted longer (12 weeks) and another case of non-union. 

Evaluating the pattern of humeral fractures and aiming for a minimally invasive and quick fixation solution, EIN was chosen as a definite treatment method. Excellent clinical, radiological, and functional results, in a follow-up period of up to 55 months, confirmed the effectiveness of elastic intramedullary nailing in humeral fractures treatment. An important aspect of this procedure that should be highlighted is the ease of nail removal (either after fracture healing or before plating in case of non-union) as well as its cost-effectiveness, cheaper than humeral nails or implants of internal fixation, quick surgery time, no requirement of surgery in case of implant removal.

To answer the question as to whether our satisfying result is an exception or validates a successful treatment method, we reviewed the literature (AA and AS). Clinical trials, case reports, and retrospective studies were searched in databases of PubMed, Embase, and Central up to December 28, 2019, using keywords “adult humerus fracture, elastic nails”. Studies including other types of fixation than elastic nails or patients younger than 18 years of age, were excluded. The use of EIN only for proximal humerus fracture was also an exclusion criterium. We ended up with seven studies that fulfill our criteria (Table 1) [14-20].

**Table 1:** Previous studies that coped with elastic humeral nails in adults ROM: Range of motion; ASES: American Shoulder and Elbow Surgeons Standardized Shoulder Assessment Form; DASH: Disabilities of the arm, shoulder, and hand.

Study (year)	Patients number (Male:Female)	Mean Age (years)	Fracture healing rate (at 4th month postoperatively)	Functional Result	Mean Follow-up time
Alshammari et al. (2019) [14]	2 (1:1)	64	2/2	Full painless ROM	18 months
Haq et al. (2012) [15]	30(21:9)	33,5	23/30	Acceptable ROM	4 months
Kornah et al. (2017) [16]	28(19:9)	29	26/28	Almost excellent (25/28)	20 months
Patel et al. (2018) [17]	20(15:5)	32,75	18/20	Excellent ASES score	20 months
Pedrazzini et al. (2019) [18]	3(0:3)	71,33	2/3	Some limitations of ROM	11 months
Upadhyay et al. (2017) [19]	25(17:8)	39,08	25/25 (8 months post-op)	22/25 excellent functional results	20 months
Verma et al. (2017) [20]	20(17:3)	38	10/20 (4 non-unions)	14/20 Normal DASH score	6 months

The complications referred to in the above studies included non-union (Haq et al. 7/30, Kornah et al. 2/28, and Verma et al. 4/20) and infections (Haq et al. 9/30, Kornah et al. 3/28, and Verma et al. 2/20) [15,16,20]. Nail impingement and elbow stiffness six months post-operatively, were present only in five patients in total, and they were the result of improper surgical technique and rehabilitation. The overall functional result was at least good in 107 of 129 patients (rate 83%). These results support our findings and encourage the use of EINs as a definite treatment for humeral fractures in cases that a minimally invasive and where fast fixation method is required.

Of course, there are cases in which the proposed technique cannot be applied. The inability of closed reduction, multi-fragmentation, and osteoporotic, fragile bone are factors that may complicate surgical procedures seriously. On the other hand, the medullary canal’s obstructions or small diameter, radial nerve impairment, intra-articularly extended fractures, and active infection at the surgical site are absolute contraindications for using intramedullary nails.

Contraindication could be an obstructed medullary canal (too narrow or too short), a radial nerve palsy (nerve possibly interposed between fracture fragments), and articular involvement. Other cases where this technique is contraindicated are extreme osteoporosis and active infection.

However, our study has significant limitations. Obviously, one case is not enough for extracting safe conclusions. A large number of parameters including fracture type and location, age of the patient, bone quality, associated injuries, surgical technique, and rehabilitation protocol make the conduction of randomized clinical trials necessary before the use of EINs can become a mainstream recommendation for humeral fractures treatment. Moreover, there is no standard tool for the evaluation of functional results according to the needs of each individual make.

## Conclusions

The use of elastic titanium intramedullary nails for multi-segmental, complex humeral fractures in adults, could be considered by an orthopedic surgeon (it can be used as primary treatment in polytrauma patients, or even as definite treatment such as in the case presented). The surgical technique of elastic intramedullary nailing is minimally invasive, time-saving, and without blood loss. Complications of additional tissue trauma by surgical approach as in plate fixation (such as atrophic non-union and radial nerve palsy) are minimized. Small learning curve is required, and it seems that satisfactory clinical and radiological outcomes with earlier restoration of function can be achieved. Further studies are required to delineate the potential of adopting elastic titanium nails for humeral fractures in standard orthopedic practice. Using elastic titanium intramedullary nails for complex and demanding humeral fracture could be a minimally invasive technique, especially in the case of polytrauma patients with life-threatening conditions. With the use of a splint for four weeks, this fixation could be the definitive treatment with excellent functional results even after five years of follow-up. There were no clinical or imaging findings of shoulder and elbow arthritis. Under extreme conditions (polytrauma patient, patient with comorbidities) an orthopedic surgeon could use this type of fixation with good results, as proved by this case and literature review.
